# Diffractive dipolar coupling in non-Bravais plasmonic lattices†

**DOI:** 10.1039/d0na00095g

**Published:** 2020-02-11

**Authors:** David Becerril, Omar Vázquez, Diego Piccotti, Elizabeth Mendoza Sandoval, Tiziana Cesca, Giovanni Mattei, Cecilia Noguez, Giuseppe Pirruccio

**Affiliations:** Instituto de Física, Universidad Nacional Autónoma de México Apartado Postal 20-364 México D.F. 01000 Mexico pirruccio@fisica.unam.mx; Department of Physics and Astronomy, University of Padova Via Marzolo 8 I-35131 Padova Italy

## Abstract

Honeycomb plasmonic lattices are paradigmatic examples of non-Bravais lattices. We experimentally measure surface lattice resonances in effectively free-standing honeycomb lattices composed of silver nanospheres. By combining numerical simulations with analytical methods, we analyze the dispersion relation and the near-field properties of these modes along high symmetry trajectories. We find that our results can be interpreted in terms of dipole-only interactions between the two non-equivalent triangular sublattices, which naturally lead to an asymmetric near-field distribution around the nanospheres. We generalize the interaction between the two sublattices to the case of variable adjacent interparticle distance within the unit cell, highlighting symmetry changes and diffraction degeneracy lifting associated to the transition between Bravais and non-Bravais lattices.

## Abstract 2

Honeycomb plasmonic lattices are characterized by a 2-particle unit cell. The difference between the intrasublattice and intersublattice coupling is distinctive of non-Bravais lattices. Although the two particles are identical the two types of coupling may be different.

## Graphical abstract

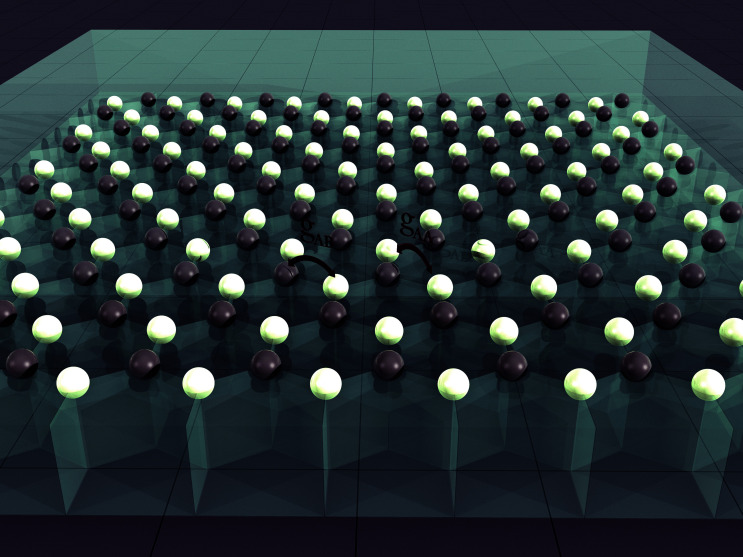

## 1 Introduction

Within the vast field of photonics, ordered structures have always received special attention. Currently, they are at the heart of many new fields of investigation intimately related to symmetry, such as topological,^1–4^ non-reciprocal,^5–7^ and PT-symmetric photonics,^8–10^ and helped in demonstrating fascinating effects including one-way light propagation,^11–14^ spin–orbit coupling^15,16^ and optical edge states.^17–19^

During the last decade the experimental investigation on plasmonic lattices, two dimensional ordered arrays of coupled metallic nanoparticles, has grown considerably.^20–22^ Plasmonic lattices sustain collective, hybrid plasmonic-photonic modes, known as Surface Lattice Resonances (SLRs) arising from the long-range, enhanced radiative coupling of localized plasmons of the individual nanoparticles.^23–28^ To a certain extent, these modes represent the optical counterpart to the electronic Bloch modes found in atomic crystals.^29,30^ Analogously, their dispersion is best studied along high symmetry trajectories within the first Brillouin zone. High symmetry points show degeneracy of the optical bands and play an important role in determining the characteristics of the near-field.^28,31^ So far, the great majority of the studies have focused on simple geometries, typically realized by Bravais lattices. Despite this, extremely rich physics and unexpected phenomena, such as plasmon^32–34^ and polariton lasing,^35,36^ strong light-matter coupling^37–40^ and quantum phase transitions,^41–43^ have emerged, making this research area vibrant and fast-growing.

After many important studies devoted to the understanding of how particle parameters and interparticle distance affect the SLR dispersion, the attention is now shifting towards uncovering the role of the lattice symmetry and complexity of its unit cell. In conventional square and rectangular Bravais plasmonic lattices, the large degree of symmetry results in many degenerate optical bands. Inspired by the recent development in material science and the ground-breaking discovery of a new class of two-dimensional non-Bravais materials, non-Bravais plasmonic lattices started receiving attention.^31,44–48^ Even though the equations governing atomic and optical lattices are different, analogies can be drawn based on translation invariance and Bloch theorem. The attractive physical properties of graphene and transition metal dichalcogenides trace back to their crystalline honeycomb structure and the presence of non-equivalent K points in the reciprocal space.^49^ Likewise, remarkable optical properties of analogous plasmonic lattices may be envisaged upon achieving exquisite control over the unit cell.

The improved nanofabrication capabilities given by electron beam and optical lithography allowed realizing nearly defect-free nanostructures, a crucial ingredient to achieve a strong collective behavior. Even though the fabrication of complex, non-Bravais lattices remains challenging, nanosphere lithography offers a relatively easy and cheap way to naturally obtain honeycomb plasmonic lattices. This technique was first introduced as a means to obtain large-scale SERS substrates,^50,51^ but the presence of defects hampered the quantitative study of plasmonic properties.^52^ Lately, advances in the self-assembly process improved the quality of the optical resonances and several properties could be investigated over large areas, including third-order optical nonlinearity,^53–56^ SERS,^57^ biosensing^58^ and modified spontaneous emission of atoms weakly coupled to the lattice.^59,60^ Interestingly, the importance of fabricating large plasmonic lattices has been recently stressed by a theoretical study on the relation between the number of unit cells and near-field collective properties.^61^

In this manuscript, we experimentally measure the dispersion relation of SLRs in honeycomb plasmonic lattices fabricated on cm-scale by nanosphere lithography, with special attention on the Γ − M trajectory of the *k*-space and s-polarization (results for the Γ − K trajectory and p-polarization are shown in the ESI†). Our lattices show a remarkable spatially homogeneous extinction which is interpreted with the help of numerical simulations and analytical calculations based on the generalized spectral representation.^62^ The observed modes result from the long-range coupling of dipole moments associated with each nanosphere. Based on our model, we propose an alternative way to evaluate the number of unit cells needed to ensure convergence of both far- and near-field response of our non-Bravais lattice. Importantly, we are able to disentangle the intra-sublattice from the inter-sublattice interaction within the 2-particle unit cell lattice and analyze them separately. The inter-sublattice interaction represents the real distinctive property of non-Bravais lattices over simple Bravais lattices commonly studied in literature. The presence of two non-equivalent sublattices allows the interaction of the lattice modes associated with each of them, which was recently related to a hierarchically higher degree of hybridization.^47^ Remarkably, our dipolar SLRs display very similar far-field and near-field characteristics shown in similar lattices with larger nanoparticles, without the need to invoke multipole moments (in the ESI† we extend our model to quadrupole interactions). The relevance of the dipolar interaction in honeycomb lattices, even in the linear regime, was also recently stressed by Kolkowsky and Koenderink.^63^ Finally, we theoretically explore the optical response of two-particles unit cell lattices beyond the honeycomb symmetry. We show that the relative translation of the two triangular sublattices leads to a smooth transition between a Bravais, rectangular lattice and a non-Bravais, honeycomb lattice with consequent point group symmetry change and degeneracy lifting of diffracted orders.

## 2 Results and discussion

**Fig. 1:**
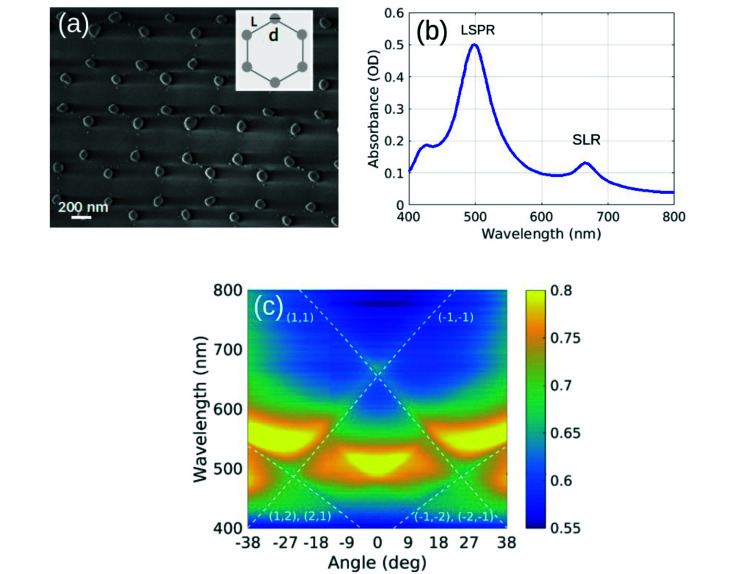
(a) SEM image of the honeycomb plasmonic lattice. (b) Measured absorbance spectrum of the lattice. (c) Measured s-polarized extinction as a function of the wavelength and angle of incidence along the Γ − M trajectory. Dashed lines indicate RAs calculated with an effective refractive index of 1.47.

We fabricated two-dimensional, honeycomb plasmonic lattices on a large scale by means of nanosphere lithography.^53^ First, we deposited a colloidal, self-assembled monolayer of 518 nm-diameter polystyrene nanospheres on a silica substrate. Then, we performed thermal evaporations of Ag followed by mechanical removal of the polystyrene nanospheres, obtaining a honeycomb lattice made of isolated, 72 nm-tall, triangular nanoprisms. By thermal annealing of the samples at 120 °C for 1 h, the metallic nanoprisms acquired a quasi-spherical shape with diameter, *d*, and center-to-center distance, *L*, equal to 100 nm and 300 nm, respectively. A SEM image of the plasmonic lattice is shown in Fig. 1(a). The small quantity of residual material observable between the nanospheres turns out to be unimportant as for the optical properties of the lattice. Finally, we sputtered a 170 nm-thick silica layer on top of the plasmonic lattice. The sputtered silica layer function is twofold: first, it avoids oxidation of the silver nanoparticles, and second, because its refractive index is very close to the one of the substrate, it creates an effectively free-standing lattice, favoring the propagation of SLRs.^20,25,64^ We note that our SEM image is taken directly from the sample used in the investigation. Therefore, some shadowing effect due to the charging of the dielectric substrate is expected.

### 2.1 Collective modes in honeycomb lattices

In Fig. 1(b) we plot the experimental spectral absorbance of the final structure, measured with a commercial Jasco V670 dual-beam spectrophotometer and calculated as −log_10_(*T*), where *T* is the normalized transmittance. To identify the three observed peaks, we measure the s-polarized extinction as a function of the wavelength and angle of incidence along the Γ − M trajectory, collected from approximately a 2 mm-spot. In Fig. 1(c) we observe several sharp lines associated with Rayleigh anomalies (RAs), *i.e.*, diffracted orders grazing to the lattice plane, calculated using an effective refractive index equal to 1.47. The main feature, around *λ* = 500 nm and 0 deg, is the localized surface plasmon resonance (LSPR) associated to the individual nanospheres. This rather non-dispersive mode couples to the (1, 1) and the degenerate (−1, −2) and (−2, −1) RAs around 27 deg, resulting in a broad, flat SLR. This mode becomes strongly dispersive and its linewidth narrows towards small angles. At normal incidence, the degeneracy between the (±1, ±1) RAs gives rise to a sharp peak around *λ* = 660 nm. The sharpness of this peak is determined by the crossing of the RAs and the wavelength detuning of the LSPR. Because of its large detuning from the main RAs, the rather weak quadrupolar resonance observed around *λ* = 400 nm in Fig. 1(b) does not play a major role in the response of our lattice. Extinction measurements for p-polarized incident light along Γ − M and Γ − K trajectories are shown in Fig. S4 in the ESI.†

**Fig. 2:**
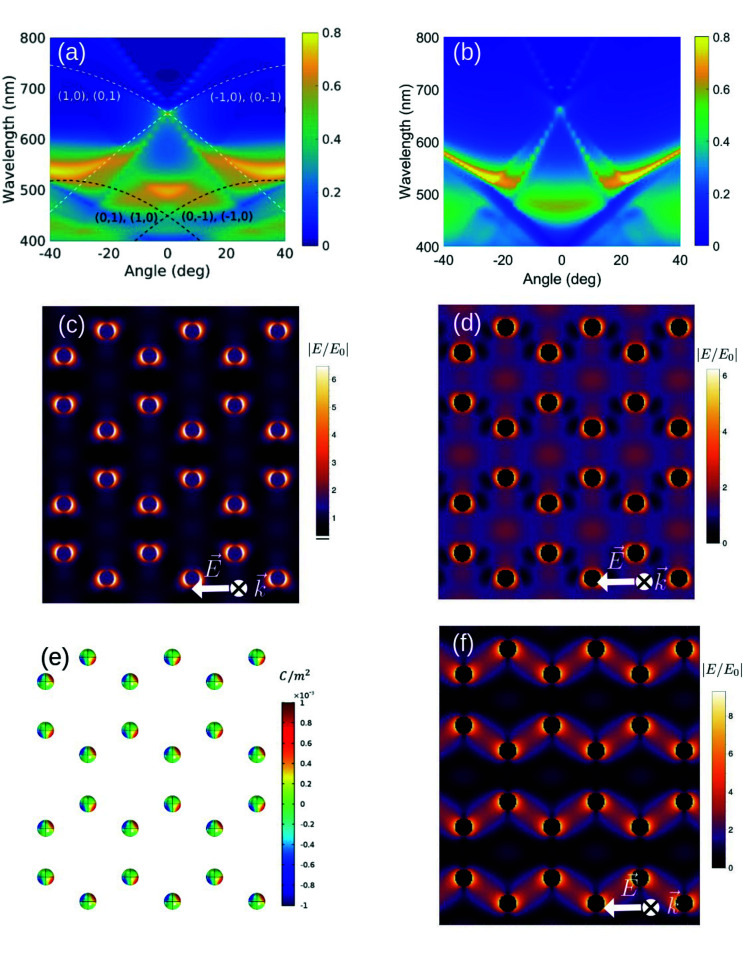
(a) Finite element method simulation and (b) spectral representation calculation of the s-polarized extinction map along the Γ − M trajectory. Dashed curves in (a) indicate RAs. (c) Simulated and (d) calculated spatial distribution of the normalized electric field amplitude for the LSPR, around *λ* = 500 nm. (e) Simulated surface charge density and (f) calculated spatial distribution of the normalized electric field amplitude for the SLR peak at *λ* = 660 nm. (c–f) are plotted in the plane crossing the nanospheres along their diameter, at normal incidence and for s-polarized incident light.

To identify the symmetry of the modes and their near field spatial distribution, we perform both electrodynamical simulations and analytical calculations. First, we calculate the scattering efficiency of an isolated, 100 nm-diameter, silver nanosphere immersed in a uniform environment with a refractive index equal to 1.45, similar to the one of silica. We compare Mie theory with the spectral representation method restricted to the dipole approximation (see Fig. S8 in the ESI†). The excellent agreement between the two methods demonstrates that the response of our nanoparticles is predominantly dipolar. The simulated and calculated s-polarized extinction maps along the Γ − M trajectory are displayed in Fig. 2(a) and (b), respectively. The simulated map is obtained by the finite element method using the commercial software Comsol Multiphysics. On the other hand, the calculated map is obtained by using a modified spectral representation method which allows taking into account multipole moments associated with each particle, multipole incident field and multipolar interaction between the nanoparticles.^62^ The main advantage of this method is the possibility of including, rather simply, multipole effects when needed. Furthermore, it provides a clear interpretation and permits a systematic analysis of the interaction between particles belonging to the same sublattice and to different sublattices. Here, considering the large interparticle distance compared to the diameter of the nanospheres, and the results shown in the ESI,† we restrict both the polarizability of the nanospheres and the Green's function describing their interaction, to be dipolar. The spectral representation is then modified to include long-range radiative terms.^65^ This modification permits the radiative coupling between the nanospheres, responsible for the excitation of the SLRs.^25^ The theoretical maps are in reasonable agreement with each other and with the experimental measurements shown in Fig. 1(c). The former confirms the assumptions made in the calculations, while the latter demonstrates that the response of our honeycomb lattice is mainly dipolar. In the theoretical maps, we recognize the SLRs associated with the RAs experimentally observed in Fig. 1(c), together with extra RAs associated to the (0, ±1) and (±1, 0) orders (dashed, white curves). The broadening of the associated extra SLRs, and hence their reduced visibility in the experiment, may be attributed to the presence of defects in the lattice and to the not-perfectly spherical shape of the nanoparticles. Nonetheless, the overall good agreement between measurements and theoretical predictions is a clear indication that the random differences in shapes are averaged out by the illumination field and that our fabrication technique is sufficiently accurate to produce samples with controlled properties. We also generalized our model to include dipole-quadrupole and quadrupole–quadrupole interactions and calculate the extinction map and the near field spatial distribution for the localized mode and the SLR. Due to the large detuning of the RAs from the quadrupole mode, we find that its contribution to the SLR is negligible. These results are shown in Fig. S1–S3 of the ESI.†

The differences between Fig. 2(a) and (b) are due to the presence of the air–silica interface, not taken into account in our calculation, that permits the propagation of RAs associated to a refractive index close to 1 (dashed, black curves). These modes, observed also in the experiment, spectrally overlap with the LSPR around 500 nm, causing its redshift and increased extinction around normal incidence. At large angles, these RAs cause the broadening and the flattening of the dispersion of the SLRs excited around *λ* = 550 nm. The role of the interface has been checked by simulating the system without the air–silica interface, obtaining an excellent agreement between the theoretical maps (compare Fig. 2(b) with Fig. S6 in the ESI†). Finally, the difference in intensity observed for *λ* > 670 nm between the modes associated to the (1, 1) and the degenerate (1, 0), (0, 1) RAs (as well as their symmetric counterparts) is due to the combination of the lattice orientation and polarization of the incident light, which determines the preferential direction of the radiation of the dipoles.^48^

Besides the far-field extinction, our analytical method allows us to calculate near-field quantities which give insights into the type of interactions present in the honeycomb lattice. Fig. 2(c) and (d) compare the simulated and calculated spatial distribution of the normalized electric near-field amplitude plotted in the plane crossing the nanospheres along their diameter. Both refer to the LSPR excited at normal incidence with s-polarized light. Apart from the small redshift induced by the interface in the simulation, we observe an overall excellent quantitative agreement in the field amplitude and spatial distribution (see Fig. S11 in the ESI†). Because of the absence of diffracted modes at this wavelength and angle of incidence, and because of the large interparticle distance, the dipoles induced in any two adjacent nanospheres do not couple to each other, and the field remains confined around each nanosphere, as expected.

Fig. 2(e) and (f) display the simulated surface charge density and the calculated spatial distribution of the electric near-field intensity of the SLR (*λ* ≈ 660 nm), at normal incidence and for s-polarized light, respectively. Calculations show that the field enhancement spreads over the lattice and in between the nanospheres, typical of the SLR. The simulated field distribution, shown in Fig. S7 in the ESI,† is in excellent agreement with the calculated one. This accord further stresses the dipolar character of the collective response of our honeycomb lattice. Noticeably, we observe an asymmetric surface charge density and electric field distribution around the two nanospheres of the unit cell, which, given our dipolar model, we interpret as the result of the dipolar interaction between the two non-equivalent triangular sublattices. Differently from the localized resonance (Fig. 2(c) and (d)), dipoles associated to non-equivalent lattice points of adjacent unit cells are coupled together *via* the standing wave resulting from the interference of the counterpropagating (±1, 0) and (0, ±1) RAs. Consequently, the maxima of the surface charge density and field amplitude are displaced with respect to the nanosphere equator. Since in these calculations the quadrupole modes of the nanospheres are not included, the hierarchical hybridization cannot explain the asymmetric near field distributions observed within the unit cell.^47^ Thus, at least in our case, such mechanism can be considered as weak. Calculations of the p-polarized SLR are in very good agreement with what is reported in ref. 47 (see Fig. S8 in the ESI†). The symmetric intensity pattern seen in the lattice plane and the maximum at the center of each hexagon result from the interference of the degenerate (±1, ±1), (0, ±1) and (±1, 0) RAs at normal incidence (see Fig. 2(a)).

An essential characteristic of the SLRs is their propagation length. This quantity relates to the number of unit cells needed to correctly describe the collective response of the plasmonic lattice. To estimate this number in our model, we consider an infinite lattice and we introduce in the Green's function a cut-off radius around each lattice point, which determines the number of interacting unit cells. Then, we vary the value of the radius until convergence of both far-field and near-field lattice response is reached. We find that a cut-off radius of 18 μm, corresponding to approximately 20 unit cells, is needed. This value is in agreement with reported experimental values.^26^

### 2.2 Intersublattice and intrasublattice coupling

**Fig. 3:**
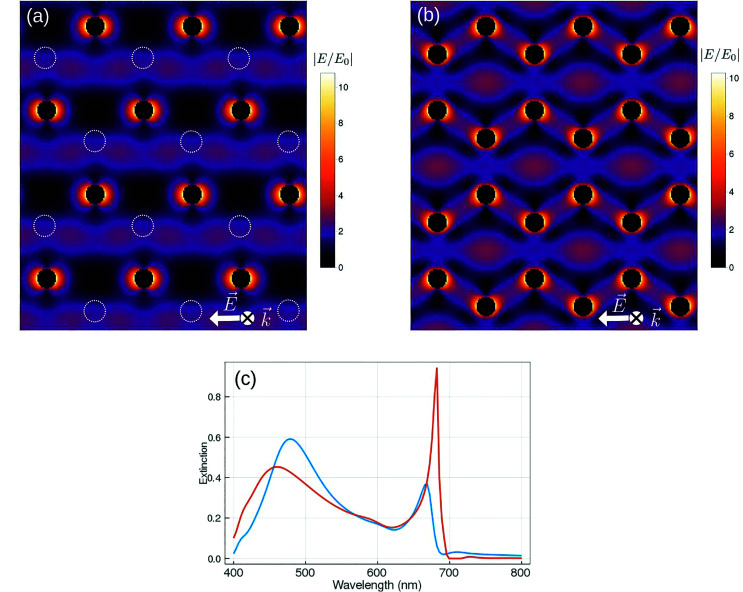
Calculated spatial distribution of the normalized electric field amplitude for (a) one triangular sublattice and (b) two superposed triangular sublattices for the SLR. The calculations are for s-polarized light and normal incidence. The dashed circles in (a) represent the position of the missing non-equivalent sublattice. (c) s-polarized extinction spectra calculated at normal incidence for the two non-interacting triangular lattices (orange curve) and for the honeycomb lattice (blue curve).

The interaction between the two triangular sublattices determines the collective response of the honeycomb plasmonic lattice. Each sublattice sustains SLRs resulting from the long-range, dipolar coupling of the corresponding nanospheres. The dispersions of these SLRs are identical since the two triangular sublattices share the same reciprocal space. However, the relative translation of one sublattice with respect to the other causes the interference of the two SLRs at the near-field level. A significant advantage of our analytical method is that it allows analyzing the contribution to the honeycomb lattice response of the two triangular sublattices. This gives new physical insight that cannot be obtained, for example, by numerical simulations, as it allows a separation of the dielectric and geometrical properties of the system. For example, by calculating the interaction matrix it is possible to analyze the coupling between the two triangular sublattices (see ESI†). Our system is tuned in such a way that, for the SLR at normal incidence, dipole–dipole interaction between particles of the same sublattice is found to be of the same order than that between particles belonging to different sublattices even though the separation distance is larger between particles belonging to the same sublattice. Furthermore, dipolar interaction is nearly four orders of magnitude larger than that of the quadrupole. This is a consequence only of the symmetry of the system and it is independent from the dielectric properties of the particles. It is instructive to calculate the spatial distribution of the near-field intensity of the SLR associated with one sublattice (see Fig. 3(a)). By using the superposition principle, in Fig. 3(b) we calculate the total near-field of the two superposed sublattices. For the SLR wavelength, we see in Fig. 3(a) that the nanospheres belonging to the missing non-equivalent sublattice fall very close to the maximum of the standing wave generated by the (±1, ±1) RAs, confirming the coupling role of these diffracted orders. This method, although providing a near-field distribution which closely resembles the one in Fig. 2(f), does not adequately take into account the coupling between the two sublattices. It corresponds to take the off-diagonal submatrices in the tensorial form of the Green's function equal to 0 (see ESI† for the details of the model). These terms, responsible for the coupling between the two sublattices, carry the information about the form factor associated with each of them. The form factor depends on the exact relative position of the sublattices and it determines the response of the honeycomb lattice. The effect of the intersublattice coupling is analyzed in Fig. 3(c), in which we compare the normal incidence extinction spectrum of the honeycomb lattice with the one of two non-interacting triangular lattices. The intersublattices coupling causes the broadening and the blueshift of the SLR peak.

### 2.3 Beyond the honeycomb lattice

**Fig. 4:**
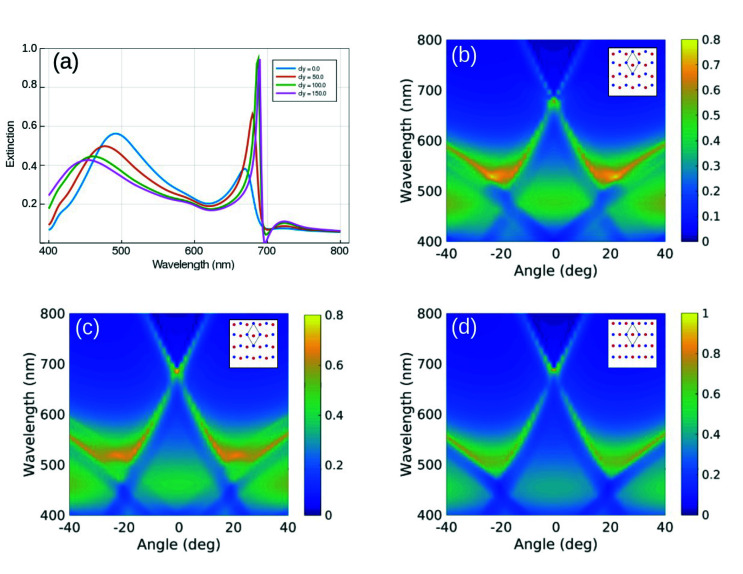
(a) s-Polarized extinction spectra calculated at normal incidence for different Δ*y*. Calculated s-polarized extinction maps for (b) Δ*y* = 50 nm, (c) Δ*y* = 100 nm and (d) Δ*y* = 150 nm, along the Γ − M trajectory. Insets show the unit cell of the non-Bravais lattices.

In Fig. 4(a) we calculate the s-polarized extinction spectrum at normal incidence of non-Bravais lattices for several values of the relative distances of the two nanospheres within the unit cell (see inset). As the interparticle distance gets larger, a transition between a honeycomb non-Bravais (Δ*y* = 0) and a rectangular Bravais lattice (Δ*y* = 150 nm) is observed. Interestingly, the LSPR blueshifts, broadens and dims, while the SLR redshifts, narrows and increases. The s-polarized extinction maps corresponding to Δ*y* = 50, 100, 150 nm are displayed in Fig. 4(b), (c), (d), respectively. For large Δ*y* we see a blueshift of the high order RAs. This causes a further increase in their detuning from the LSPR at normal incidence. Consequently, it lowers the LSPR extinction and sharpens the SLR. For the honeycomb lattice, the wavevectors of the two pairs of counterpropagating RAs (±1, 0) and (0, ±1) associated with each sublattice form an angle of 30 degrees with the electric dipoles of the nanospheres located along their propagation directions. On the contrary, for the rectangular lattice, the two counterpropagating RAs, (0, 1) and (0, −1), have wavevectors perpendicular to the electric dipole associated to each nanosphere. This maximizes the coupling between them and the excitation efficiency of the SLR at normal incidence (see Fig. 4(a) and S9 in the ESI†). Moreover, intriguing features which modify the SLR dispersion, appear around 25 deg. The relative translation of the two particles within the unit cell cannot influence the intrasublattice interaction. Thus, this cannot be the origin of the dispersion modification. On the other hand, the intersublattice interaction term of the Green tensor depends on the phase associated to the form factor of the lattice. This is a characteristic of non-Bravais lattices and stresses the uniqueness of bipartite systems over the conventional single-particle unit cell lattices, providing an extra degree of freedom to tailor the near-field properties and the dispersion of the modes of this system. From the symmetry point of view, the larger excitation efficiency of the SLR results from considering the transition between the non-Bravais and Bravais lattice, which is continuous at normal incidence. The latter is accompanied by the change in the point group, which implies a lower rotation order and, thus, a lower number of degenerate RAs at normal incidence for the rectangular lattice.^48^ Specifically, this means that for the rectangular lattice and s-polarization, (i) the non-degenerate (±1, 0) RAs, active in the honeycomb lattice, become forbidden, and (ii) the (±1, ±1) RAs shift considerably in wavelength and do not contribute anymore to the SLR excitation. In the ESI,† we analyze the effect of *x*-translation of the two triangular sublattices (see Fig. S10†).

## 3 Conclusions

Non-Bravais plasmonic lattices exhibit complex, multiparticle unit cells which offer extra degrees of freedom to tune both near and far-field lattice response. We fabricated and experimentally investigated a non-Bravais honeycomb plasmonic lattice composed of effectively free-standing silver nanospheres sustaining surface lattice resonances. Excellent agreement is found between experiments, calculations based on the spectral representation method and finite elements simulations, establishing nanosphere lithography as an effective, large-scale fabrication method. Dipolar interactions dominate the collective response of the lattice. We observe asymmetric field distribution within the unit cell, even when the particle response is dipolar. This is explained by considering the geometrical arrangement of the two non-equivalent triangular sublattices that separately sustain collective modes. Their careful relative positioning permits the transition between a non-Bravais, honeycomb and Bravais, rectangular lattice. This smooth change in the point group symmetry is related to the activation or suppression of multiple degenerate diffracted orders. The tailored phase difference between the individual lattice modes in the near-field allows fine-tuning the extinction spectrum of the full lattice and causes intriguing modifications of the mode dispersion appearing off-normal incidence. All these characteristics are entirely due to the intersublattice interaction and are well-described within our analytical model.

## Conflicts of interest

There are no conflicts to declare.

## Supplementary Material

NA-002-D0NA00095G-s001
