# Supplementary material for: Diffractive dipolar coupling in non-Bravais plasmonic lattices
Source: Nanoscale Adv. 2020 Feb 11;2(3):1261–8. doi: 10.1039/d0na00095g (PMC9417907; doi:10.1039/d0na00095g)
Supplement: NA-002-D0NA00095G-s001 [file NA-002-D0NA00095G-s001.pdf]

Electronic Supplementary Material (ESI) for Nanoscale Advances.
This journal is © The Royal Society of Chemistry 2020

Supplemental Information: Diffractive dipolar coupling in non-Bravais plasmonic lattices

D. Becerril,† O. Vazquez,† D. Piccotti,‡ E. Mendoza Sandoval,† T. Cesca,‡ G. Mattei,‡ C. Noguez,† and G. Pirruccio*,†

†*Instituto de Física, Universidad Nacional Autónoma de México, Apartado Postal 20-364, México D.F. 01000, México*

‡*Department of Physics and Astronomy, University of Padova, Via Marzolo 8, I-35131 Padova, Italy*

E-mail: pirruccio@fisica.unam.mx

1 Analytical Model

Consider a 2-d array of identical, non-magnetic metallic particles, which can be obtained through the translation of a unit cell with N particles by lattice vectors $\vec{t_1}$ and $\vec{t_2}$. The position of a particle in the lattice can be determined by

$$\vec{r}_{jmn} = \vec{r}_{j00} + m\vec{t_1} + n\vec{t_2}, \tag{1.0.1}$$

where $j = 1, ...N$ and $\vec{r}_{j00}$ is the position of the j-th particle relative to the cell, and $(m, n) = 0, \pm 1, \pm 2 \ldots$. In this work we use a the spectral representation method[1] at the dipole level, and due to the large interparticle separation distances, modify the interaction terms to

include long-range radiative terms. The method can be seen as analogous to the coupled dipole approximation.[2] The response of a particle to the local electric field at position $\vec{r}_{jmn}$ is given via its complex dipole polarizability through equation

$$\vec{p}_{jmn} = \alpha(\omega)\vec{E}(\vec{r}_{jmn}). \tag{1.0.2}$$

The electric dipole polarizability of a spherical particle $\alpha(\omega)$, can be obtained from Mie theory[3] and is taken as $\alpha(\omega) = (3ik/2)a_1(\omega)$, where $a_1(\omega)$ is the first order Mie scattering coefficient.[4] The local electric field $\vec{E}(\vec{r}_{jmn})$ is the sum of the external field and the field due to the dipoles induced on all other particles in the lattice. The latter can be written using the dipole-dipole interaction matrix,[5] which describes the field generated by a dipole at position $\vec{r}$ and felt by a particle at $\vec{r}'$, given by

$$\mathbf{G}^0(\vec{r},\vec{r}') = \frac{e^{ikR}k^2}{R}\left[\left(1 - \frac{1}{(kR)^2} + \frac{i}{kR}\right)\mathbf{I} + \left(-1 + \frac{3}{(kR)^2} - \frac{3i}{kR}\right)\hat{R}\otimes\hat{R}\right], \tag{1.0.3}$$

where $R = |\vec{r}' - \vec{r}|$. Using Eq. (1.0.3) in Eq. (1.0.2) we obtain

$$\vec{p}_{jmn} = \alpha_j(\omega)\left[\vec{E}^{ext}(\vec{r}_{jmn}) + \sum_{j'm'n'}\mathbf{G}^0(\vec{r}_{jmn},\vec{r}_{j'm'n'})\vec{p}_{j'm'n'}\right]. \tag{1.0.4}$$

where the external field is taken to be a monochromatic plane wave $\vec{E}^{ext} = \vec{E}_0\exp(i\vec{k}\cdot\vec{r} - i\omega t)$. Notice that Eq. (1.0.6) can be obtained by generalizing the spectral representation[1] in the dipole approximation to take into account the radiative terms in the dipole-dipole interaction.

Due to the periodicity of the system, Bloch's theorem guarantees that solutions to Eq. (1.0.4) must have the form $\vec{p}_{jmn} = \vec{p}_{j00}\exp\left[i\vec{k}_{\|}\cdot(m\vec{t}_1 + n\vec{t}_2)\right]$, where $\vec{k}_{\|}$ is the projection of the incident wavevector onto the lattice. Introducing this condition into Eq. (1.0.4), the number of equations is reduced to

$$\vec{p}_j = \alpha_j(\omega)\left[\vec{E}^{ext}(\vec{r}_j) + \sum_{j'm'n'}\tilde{\mathbf{G}}^0_{j,j'}\cdot\vec{p}_{j'}\right]. \tag{1.0.5}$$

where $\tilde{\mathbf{G}}^0_{j,j'} = \sum_{m,n} \mathbf{G}^0(\vec{r}_{j00}, \vec{r}_{j'm'n'}) \exp\left[i\vec{k}_{\|} \cdot (m\vec{t}_1 + n\vec{t}_2)\right]$. Solutions to Eq. (1.0.5) can be obtained by

$$\vec{p} = \frac{1}{1/\alpha - \tilde{\mathbf{G}}} \vec{E} \tag{1.0.6}$$

where $\vec{p}$ and $\vec{E}$ are $3N$ vectors and $1/\alpha$ and $\tilde{\mathbf{G}}$ are $3N \times 3N$ matrices. Notice that when the real parts of $1/\alpha$ and $\tilde{\mathbf{G}}$ are equal, the real part of the denominator in Eq. (1.0.6) goes to zero, defining the condition of a surface lattice resonances of the system.[6]

It is useful to consider the specific case of a non-bravais lattice with honeycomb symmetry, which can be seen as a superposition of two triangular lattices. Notice that matrix $\tilde{\mathbf{G}}$ will be of the form

$$\tilde{\mathbf{G}} = \begin{pmatrix} \tilde{G}(A,A) & \tilde{G}(A,B) \\ \tilde{G}(B,A) & \tilde{G}(A,A) \end{pmatrix} \tag{1.0.7}$$

where $\tilde{G}(A,A)$ is a 3×3 submatrix which describes interaction between particles of the same triangular sublattice and $\tilde{G}(A,B)$ describes interaction between particles of different sublattices.

The terms $\tilde{G}(A,B)$ can be written in the form:[7]

$$\mathbf{G}_{\alpha,\beta}(A,B) = \lim_{z \to 0} \left(\frac{2\pi i}{A_{cell}} \sum_{mn} \frac{1}{k_z^g} \left[k^2 - (\vec{g}_{mn} + \vec{k}^{\|})_\alpha (\vec{g}_{mn} + \vec{k}^{\|})_\beta\right] e^{i[(\vec{g}_{nm} + \vec{k}^{\|}) \cdot \vec{r}_{AB} + k_z^g |z|]} \right) \tag{1.0.8}$$

where $\alpha, \beta = x, y, z$, $\vec{r}_{AB}$ is the vector that joins the particles of the different sublattices, $\vec{g}_{mn} = m\vec{g}_1 + n\vec{g}_2$ is a vector of the reciprocal lattice and $\vec{g}_1$, $\vec{g}_2$ are primitive vectors of the reciprocal space, $\vec{k}^{\|}$ is the projection of the incident wavevector onto the array, $k_z^g = \sqrt{k^2 - g_{mn}^2}$ and A_{cell} is the area of the unit cell. In Eq. (1.0.8) appears explicitly the form factor providing a phase difference between the two non-equivalent sublattices. Notice that from Eqs. (1.0.5)-(1.0.8) it can be seen that the resonant condition Re $\left[1/\alpha - \tilde{\mathbf{G}}\right]$ will depend primarily on elements of submatrix $\tilde{\mathbf{G}}(A,A)$, therefore resonances will correspond to the symmetries of a triangular lattice. However, matrix elements $\tilde{\mathbf{G}}(A,B)$ can significantly alter

the coupling to the different resonant modes present in the system. Eq. (1.0.7) is equivalent to that obtained using a spectral representation in the dipole approximation,[1] with analogous resonant conditions.

2 Quadrupole Contribution

To further demonstrate the dipolar nature of our SLR and to evaluate the weight of the quadrupole mode of the individual particle, we calculated the extinction spectrum including both dipolar and quadrupolar interaction. Under this approximation, the dipole and quadrupole moments induced on each particle are calculated taking into account dipole-dipole, dipole-quadrupole and quadrupole-quadrupole interaction. Eq. (1.0.7) is modified in order to take into account these new interactions, and is of the form:

$$\tilde{\mathbf{G}} = \begin{pmatrix} \tilde{G}^{p-p}(A,A) & \tilde{G}^{p-Q}(A,A) & \tilde{G}^{p-p}(A,B) & \tilde{G}^{p-Q}(A,B) \\ \tilde{G}^{Q-p}(A,A) & \tilde{G}^{Q-Q}(A,A) & \tilde{G}^{Q-p}(A,B) & \tilde{G}^{Q-Q}(A,B) \\ \tilde{G}^{p-p}(B,A) & \tilde{G}^{p-Q}(B,A) & \tilde{G}^{p-p}(B,B) & \tilde{G}^{p-Q}(B,B) \\ \tilde{G}^{Q-p}(B,A) & \tilde{G}^{Q-Q}(B,A) & \tilde{G}^{Q-p}(B,B) & \tilde{G}^{Q-Q}(B,B) \end{pmatrix}, \tag{2.0.1}$$

where $\tilde{G}^{p-p}$ are 3×3 , $\tilde{G}^{p-Q}$ are 5×3 and $\tilde{G}^{Q-Q}$ are 5×5 submatrices that describe dipole-dipole, dipole-quadrupole and quadrupole-quadrupole interaction respectively. Notice that Eq (2.0.1) depends only on the geometrical parameters of the system, the wavevector and wavelength of the incident field. Dielectric parameters of the particles are described by the electric dipole and quadrupole polarizability,[8] and must be introduced to solve for the induced dipole and quadrupole moments.

The electric field scattered by the induced dipole and quadrupole can be written in the far field $kr \gg 1$ as:[8]

$$\vec{E}_s(\vec{k},\vec{r}) = \frac{k^2 e^{ikr}}{r} \left(\mathbf{I} - \hat{r} \otimes \hat{r}\right) \sum_j e^{-i\vec{k}\cdot\vec{r}_j} \left[\vec{p}_j - \frac{ik}{6}\left(\overleftrightarrow{Q}_j \cdot \hat{r}\right)\right], \tag{2.0.2}$$

where $\vec{p}_j$ and $\overleftrightarrow{Q}_j$ are the induced dipole and quadrupole moments on particle j, and $\mathbf{I}$ is a 3×3 identity matrix.[8] Eq. (2.0.2) is then used to calculate the extinction, making it is possible to separate the dipole and quadrupole contributions. These are shown in Fig. S1. The extinction maps calculated including quadrupole interaction and s-polarazation are also shown.

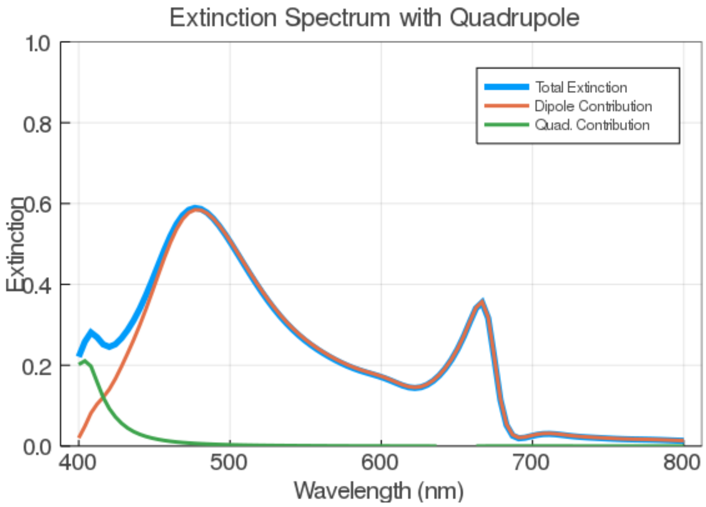

Figure S1: Extinction spectrum at normal incidence including dipole-dipole, dipole-quadrupole and quadrupole-quadrupole interaction.

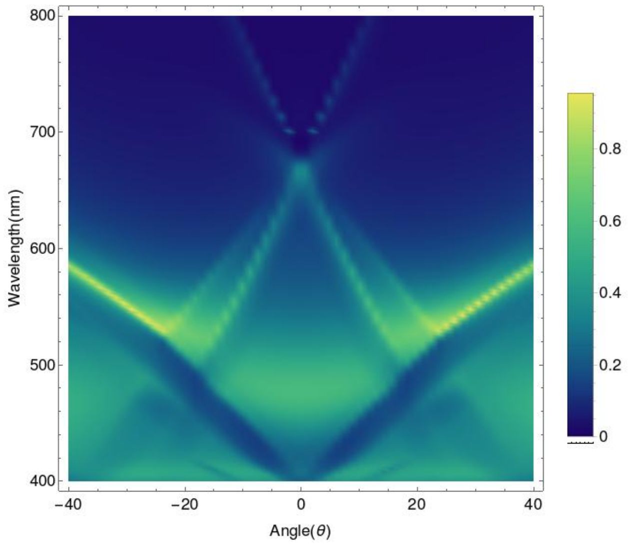

Figure S2: Extinction map for s-polarization including dipole-dipole, dipole-quadrupole and quadrupole-quadrupole interaction.

To verify that the near-field results are not significantly different when calculated under

the dipole or quadrupole approximations, we show the electric near-field calculated including dipole-dipole, dipole-quadrupole and quadrupole-quadrupole interaction. These fields are analogous to Figs. 2(d) and 2(f) of the main text. A SLR with a strong quadrupolar character can be excited by bringing a diffraction edge close to the quadrupolar resonance. However, the RAs and the SLR studied in this work are very detuned from that resonance.

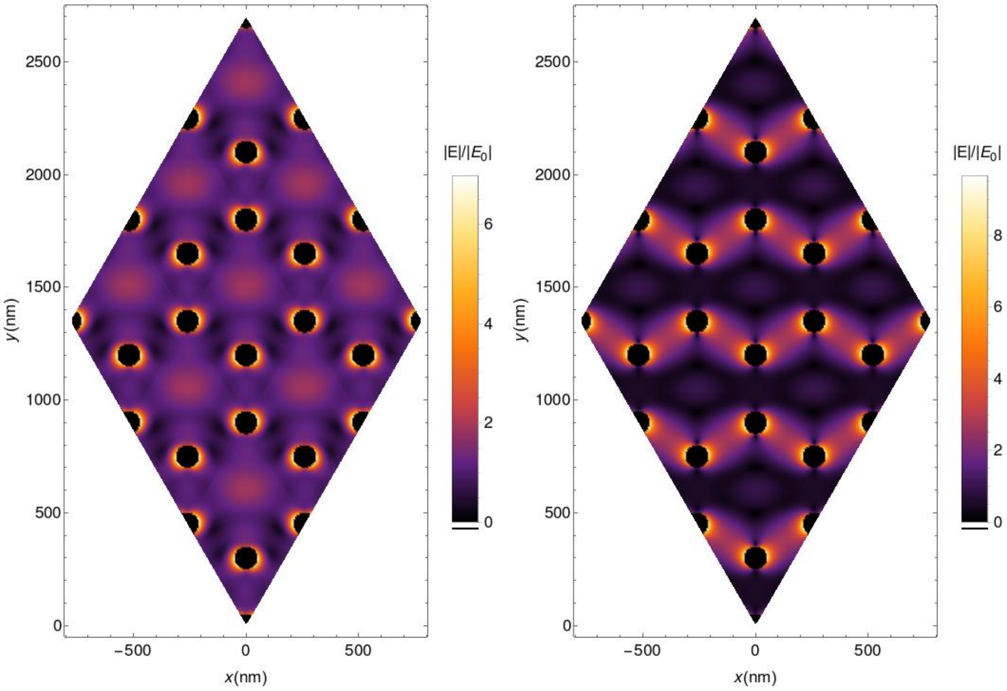

Figure S3: Electric near-field for normal incidence and s-polarization including dipole-dipole, dipole-quadrupole and quadrupole-quadrupole interaction for $\lambda = 477$ nm (left) and $\lambda = 660$ nm (right).

3 Simulations

For the numerical simulation of the extinction we used the Finite Elements Method (FEM) to solve the Maxwells equations employing the software COMSOL 5.3 with the Wave Optics package. A 3D simulation box was designed to model the extinction of a honeycomb lattice as a function of incidence angle and wavelength. We used a rhombic cell accommodating the two particles of the unit cell and we defined Floquet Periodic Boundary condition on all its sides. Port Boundary condition are defined at the top and bottom of the cell with Perfectly

Matched layers (PMLs) behind them. A user-defined plane wave illumination is performed by means of the port located on the air side of the structure. Zeroth-order transmission is evaluated with an opposite port located on the substrate side through the built-in S12 matrix element. The distance between the lattice and the ports must been large enough to avoid any evanescent field within the modeling domain. In our case the height of the substrate and air domains was of 850 nm. We checked that all our far field and near field results are independent on the type of cell used. To do this we compared with a rectangular and a hexagonal cell also using periodic boundary conditions. The former one contains 4 particles, while the latter contains 6 times 1/3 of a particle. We crosschecked that our calculation correctly treats the diffraction orders of the structure by verifying the consistency of the far and near field results when Periodic Ports and built-in wave excitation are employed.

4 Experimental and theoretical results

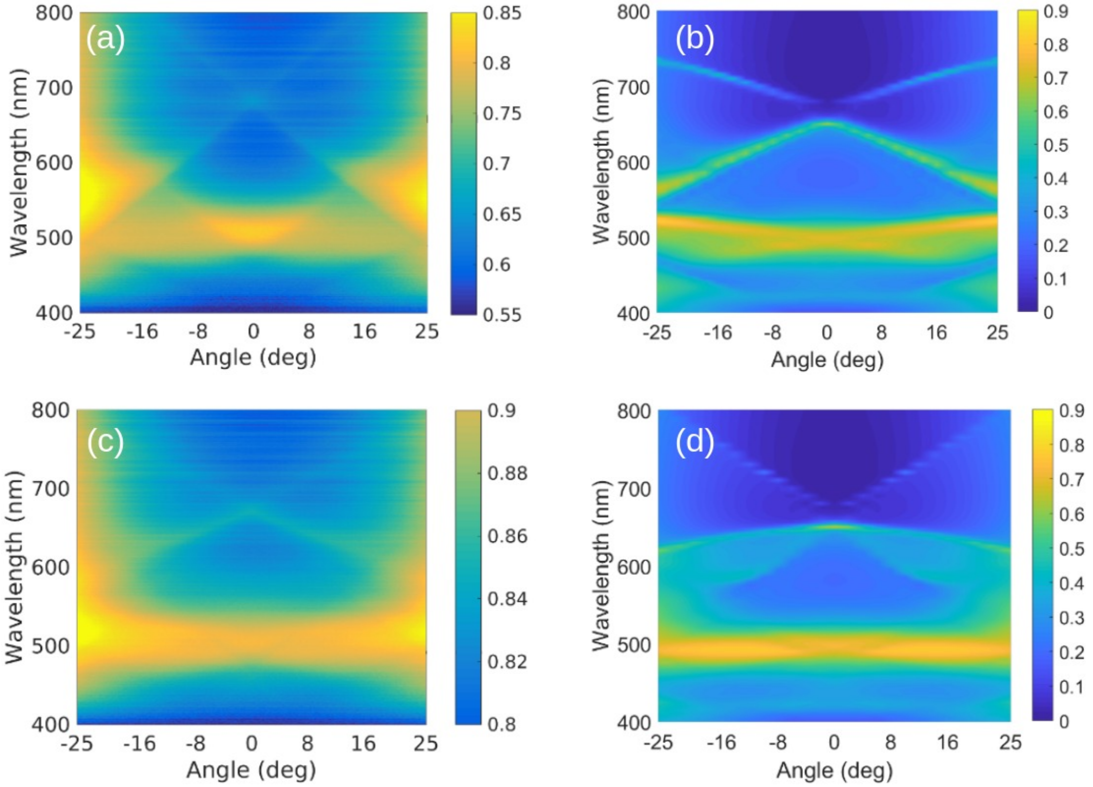

Figure S4: Measured p-polarized extinction as a function of the wavelength and angle of incidence along the (a) $\Gamma - M$ and (c) $\Gamma - K$ trajectory. Simulated p-polarized extinction as a function of the wavelength and angle of incidence along the (b) $\Gamma - M$ and (d) $\Gamma - K$ trajectory.

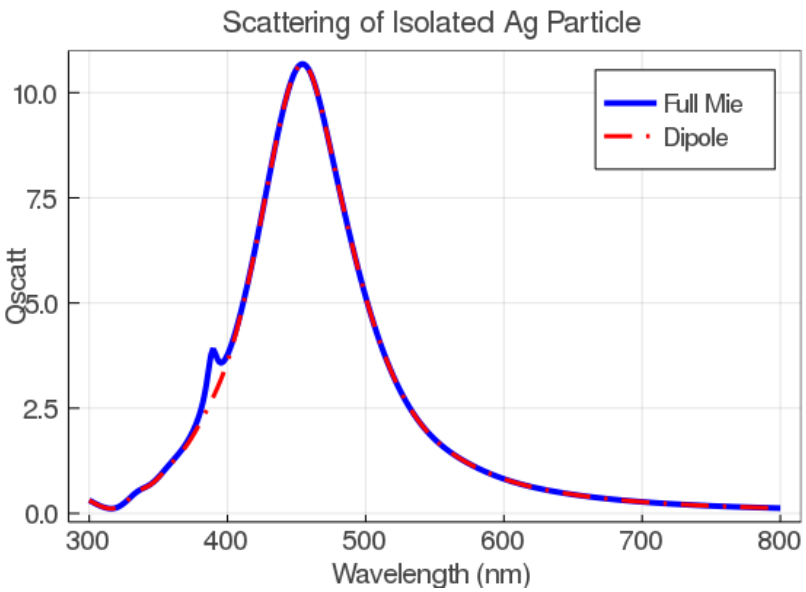

Figure S5: Cross section efficiency calculated by Mie theory (blue curve) and spectral representation method (red curve). The main peak corresponds to the dipolar localized plasmon mode of the nanosphere and it matches well with the one in Fig. 1(b). The only difference between the two calculations relates to the small peak around $\lambda = 400$ nm, which is associated to an out of plane quadrupole localized plasmon mode.[9]

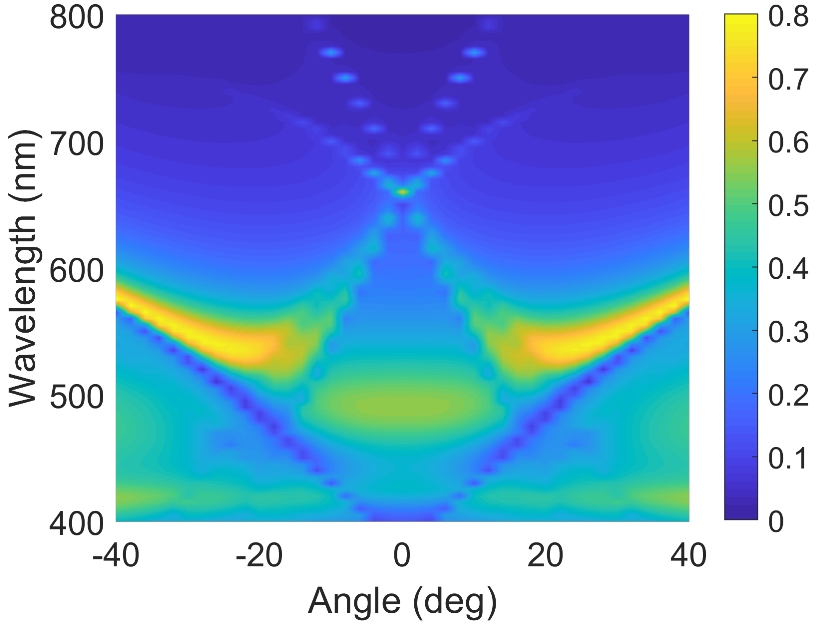

Figure S6: Simulated s-polarized extinction map, along the $\Gamma - M$ trajectory, for the honeycomb lattice homogeneously surrounded, i.e, without air-silica interface.

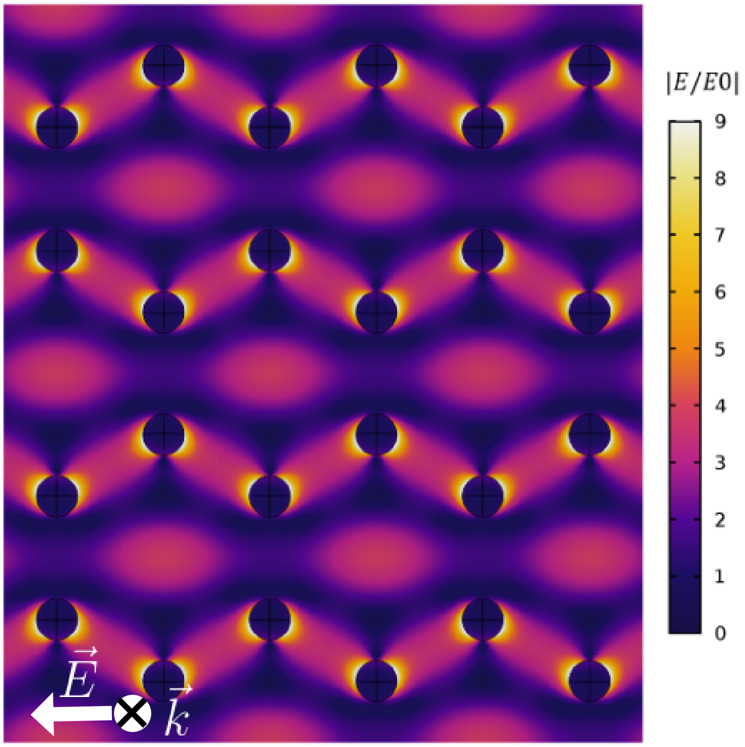

Figure S7: Simulated spatial distribution of the normalized electric field amplitude for s-polarized incident light and for the SLR.

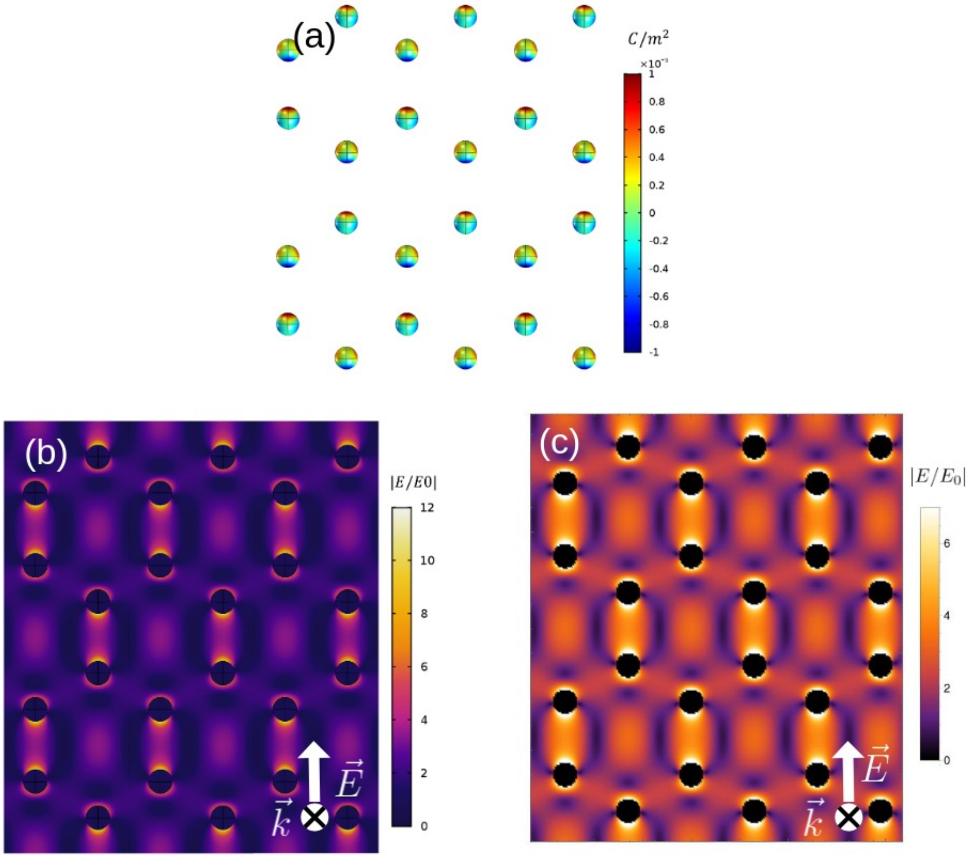

Figure S8: (a) Surface charge density simulated for p-polarized incident light, at the SLR wavelength. (b) Calculated and (c) simulated spatial distribution of the normalized electric field intensity for p-polarized incident light, at the SLR wavelength.

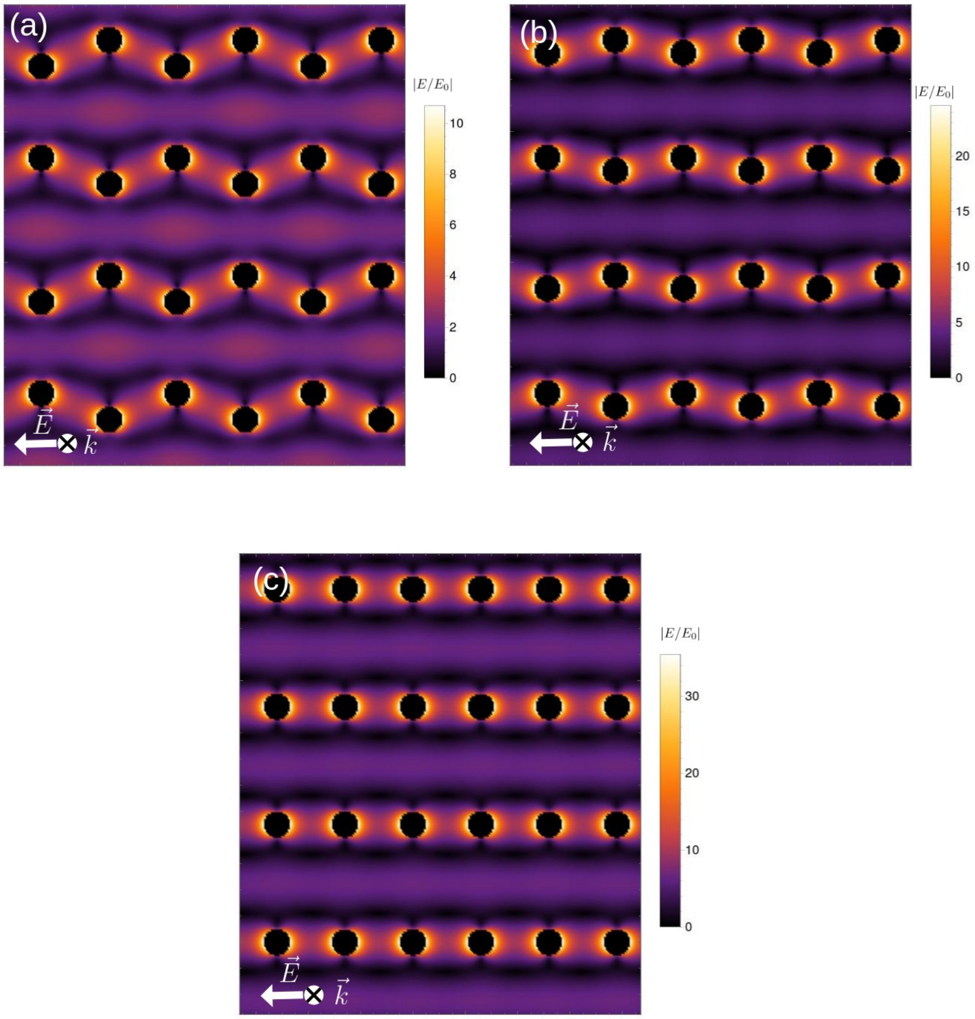

Figure S9: Calculated spatial distribution of the normalized electric field intensity for p-polarized incident light, at the SLR wavelength for the non-Bravais lattices with (a) $\Delta y = 50$ nm, (b) $\Delta y = 100$ nm, and (c) $\Delta y = 150$ nm.

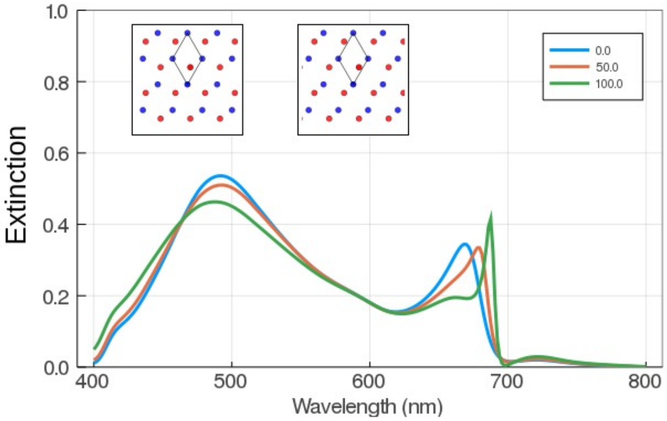

Figure S10: (a) s-polarized extinction spectra calculated at normal incidence for different Δx.

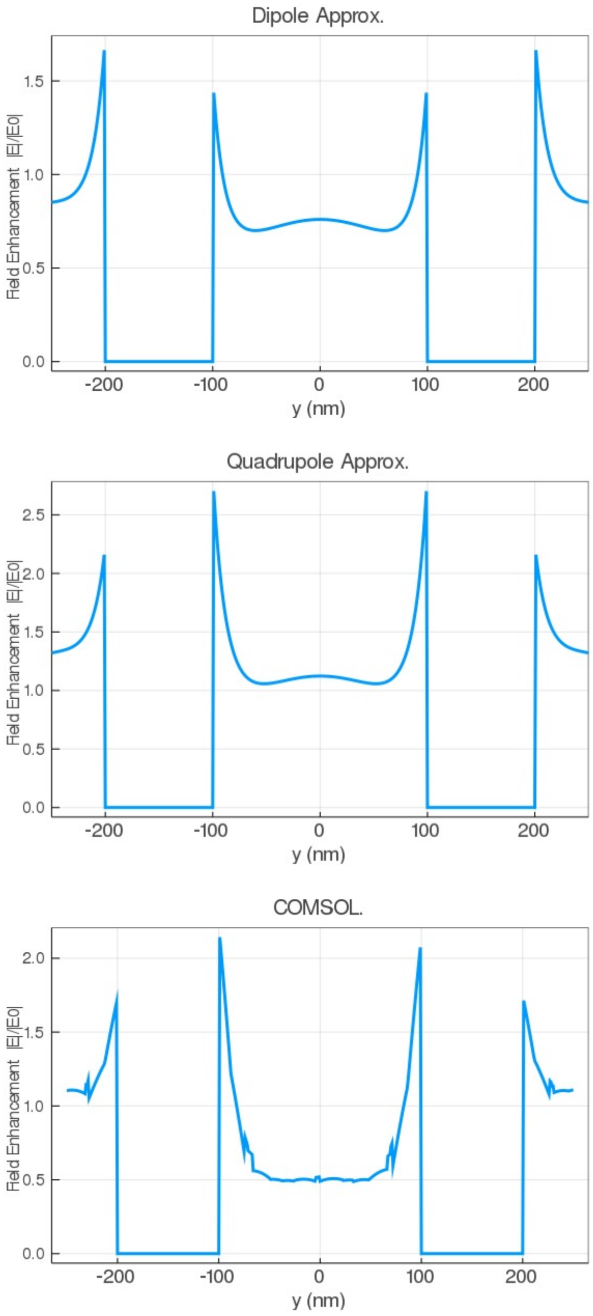

Figure S11: Cut of Fig. 2 (c) and (d) along the axis perpendicular to the the incident electric field. The regions between ±100 nm and ±200 nm corresponds to the position of the nanospheres. The middle panel is obtained after inclusion of the quadrupole interactions, which improves the agreement between the calculation and the simulation. As expected, the only difference between the results obtained under dipole and quadrupole approximation is the intensity in the close proximity of each sphere.

References

(1) Becerril, D.; Batiz, H.; Pirruccio, G.; Noguez, C. Efficient Coupling to Plasmonic Multipole Resonances by Using a Multipolar Incident Field. *ACS Photonics* **2018**, *5*, 1404–1411.

(2) Draine, B. T.; Flatau, P. J. Discrete-dipole approximation for periodic targets: theory and tests. *J. Opt. Soc. Am. A* **2008**, *25*, 2693–2703.

(3) Mulholland, G. W.; Bohren, C. F.; Fuller, K. A. Light Scattering by Agglomerates: Coupled Electric and Magnetic Dipole Method. *Langmuir* **1994**, *10*, 2533–2546.

(4) Bohren, C.; Huffman, D. R. *Absorption and Scattering of Light by Small Particles*; Wiley Science Paperback Series, 1998.

(5) Jackson, J. D. *Classical electrodynamics*, 3rd ed.; Wiley: New York, NY, 1999.

(6) Kravets, V. G.; Kabashin, A. V.; Barnes, W. L.; Grigorenko, A. N. Plasmonic Surface Lattice Resonances: A Review of Properties and Applications. *Chemical Reviews* **2018**, *118*, 5912–5951, PMID: 29863344.

(7) García de Abajo, F. J. Colloquium: Light scattering by particle and hole arrays. *Rev. Mod. Phys.* **2007**, *79*, 1267–1290.

(8) Evlyukhin, A. B.; Reinhardt, C.; Zywietz, U.; Chichkov, B. N. Collective resonances in metal nanoparticle arrays with dipole-quadrupole interactions. *PHYSICAL REVIEW B* **2012**, *85*, 1–12.

(9) Li, R.; Bourgeois, M. R.; Cherqui, C.; Guan, J.; Wang, D.; Hu, J.; Schaller, R. D.; Schatz, G. C.; Odom, T. W. Hierarchical Hybridization in Plasmonic Honeycomb Lattices. *Nano Lett.* **2019**, *19*, 6435–6441.
